# Natural selection at the cellular level: insights from male germ cell differentiation

**DOI:** 10.1038/s41418-021-00812-0

**Published:** 2021-06-02

**Authors:** Daniel H. Nguyen, Diana J. Laird

**Affiliations:** Department of Obstetrics, Gynecology and Reproductive Science, Center for Reproductive Sciences, Eli and Edythe Broad Center for Regeneration Medicine and Stem Cell Research, UCSF, San Francisco, CA USA

**Keywords:** Cell biology, Genetics

Waddington’s concept of differentiation as an epigenetic landscape provides an enduring metaphor visualizing the options faced by stem and progenitor cells. However, increasing understanding of cellular heterogeneity poses new questions about the identities and behaviors of the cells beginning this process. We now recognize a greater diversity of initial states for individual progenitor cells, which may affect their trajectories and disrupt progress entirely. Here, we consider how developmental selection occurs when heterogeneity in differentiating progenitors produces divergent cellular outcomes of survival versus elimination.

Heterogeneity is a fundamental property of biological systems. Individual cell properties like location or cell cycle can yield vastly different behaviors, and single cell analysis offers deeper characterization of transcriptional and genetic diversity. Heterogeneity in progenitors can wield lasting impacts on the composition of lineages. Variable Myc expression in epiblast cells generates selection for Myc-high populations [1] and similar enrichment occurs based on differential Hippo signaling [2]. Heterogeneous expression of P53 [3] or mTOR [4] forms the basis for clonal expansion in hematopoietic stem cells and early mouse embryos. In these examples, differentiation from heterogeneous progenitors involves cell competition and active elimination based on fitness. Importantly, competitive states are heritable across cell division, evoking principles similar to Darwinian selection. Moreover, evidence of developmental failure and selection challenges the notion that differentiation follows a robust and stable trajectory, especially at the individual cell level.

The germline is a fascinating context for investigating the consequences of heterogeneity on differentiation and cell fate. As fetal germ cells establish the gametes, their population dynamics can greatly influence inheritance. The conflict between diversity and orderly differentiation looms centrally over germline development. In mouse embryos, germ cells undertake an epic journey, from specification through sex differentiation, replete with opportunities for heterogeneity to develop and be assessed. Notably, an excess of germ cells is produced and then pruned by programmed cell death [5]. This occurs across diverse species regardless of sex, suggesting that differential fitness and elimination are critical.

Deciduous progenitor cells feature in many developmental settings, including gastrulation and neural development [6], and may be strategic for resolving heterogeneity during differentiation. In the germline, the basis for this potential selection is obtuse, although recent studies illuminate how variation emerges and affects differentiation at a more granular population level. Here, we focus on the male lineage in the fetal period, as developmental diversification and elimination during fetal oogenesis is more complex, involving meiotic entry and asymmetric nurse cell-oocyte cytoplasmic transfer [7].

In mouse fetal testes, germ cells undergo a stereotypical period of apoptosis shortly after male differentiation. This event represents a developmentally-programmed selective barrier, which we examined by characterizing apoptotic germ cells and germ cell diversity [8]. Multicolor lineage labeling revealed that dying germ cells were clonally related and shared a common cellular ancestry. This indicated that heritable factors determine distinct apoptotic fates among germ cell subpopulations. It should be noted that germ cells at this stage are connected by intercellular bridges that permit cytoplasmic exchange. These conserved germ cell structures are mitotically-produced and can reinforce clonal behavior through sharing of cytoplasmic factors between daughter cells [9]. In *Drosophila* testes, bridges can synchronize apoptosis in response to DNA damage even when only a subset of germ cells suffer insults [10]. While such connectivity resembles clonal behavior, we distinguished that clonal apoptosis in mouse fetal germ cells persists in mutants incapable of forming bridges—thereby substantiating that apoptotic fate is heritable and intrinsic.

To ascertain the basis of this apoptotic fate, we identified a male apoptosis-prone subpopulation characterized by elevated expression of pro-apoptotic genes such as Bad and p53. This transcriptional signature was associated with persistent expression of genes from a sex-undifferentiated state, whereas a reciprocal population was demarcated by male-differentiated genes and downregulation of the apoptotic signature. Together, these transcriptional profiles suggest a dichotomy between germ cell death and differentiation.

Tethering apoptotic resistance to successful differentiation may function as a quality-control mechanism. Prospermatogonial differentiation genes such as *Nanos2* also promote survival, as evidenced by elevated apoptosis in *Nanos2*^*−/−*^ [11]. Preceding male differentiation, apoptotic transcripts remain high in germ cells but diminish at the onset of *Nanos2* [8]. Thus, apoptosis can act as a differentiation fail-safe to eliminate aberrant or suboptimally differentiated cells. Coordinating apoptosis with differentiation in progenitors may prevent multipotent cells like early germ cells from tumorogenesis due to inappropriate differentiation. Similarly tight control over differentiation may direct removal of aneuploid progenitors in early embryos to prevent aberrant development and cancer [12].

What disrupts sex differentiation in certain germ cells to produce clonal elimination? An interesting link emerges from evidence for epigenetic control of sex differentiation. Germ cell sex differentiation can be modulated by interfering with DNA demethylation machinery, including Dnmt1 and Tet1; these regulate the expression of a restricted set of germline reprogramming-responsive (GRR) genes via promoter methylation [13]. Timely, efficient male differentiation relies upon activation of GRR genes by DNA demethylation and protection from aberrant methylation. We found that GRRs were hypermethylated and under-transcribed in apoptosis-prone germ cells, which likely hinders male differentiation. What might cause aberrant methylation? Early germ cell development involves extensive resetting of the epigenome for totipotency and germ cell and sex-specific identities. Such intense epigenetic remodeling in an acute period risks erroneous demethylation. These “epimutations” are far more likely to arise in progenitors than de novo genetic mutations [14] and could potentially provide a heritable, differentiation-deficient phenotype.

Epigenetic regulation of differentiation is commonplace in developing tissues including blood, skin, and intestine [15]. We predict that epigenetically-regulated gene sets analogous to GRRs control differentiation in other contexts. These loci could therefore be epimutation hotspots to generate heterogeneous differentiation potential. Single-cell methylomics promises exciting insights into the epigenetic diversity of subpopulations and their varied fates during differentiation.

With evidence that aberrant epigenetic regulation underlies the choice between differentiation and apoptosis in male-differentiating germ cells, how might this influence germ cell diversity? The heritable nature of epimutations predicts that certain clones will predominate the pool of prospermatogonia; indeed, prolonged clonal shifts in spermatogonial stem cells were detected through long-term clonal tracking [16]. By eliminating clones with epimutations in GRRs, apoptosis promotes male differentiation at the cost of potential epigenetic diversity. Under normal conditions, this may function to curtail improperly differentiated tumorigenic germ cells. However, during stress, epimutation-based phenotypes may be better tolerated to survive through gametogenesis. This is suggested by the intergenerational epigenetic inheritance of glucose-insensitive metabolic phenotypes through differential sperm methylation during starvation [17]. Progenitors with more erroneous epigenetic reprogramming may differentiate less efficiently but possess adaptive advantages. For example, male germ cell differentiation is accompanied by upregulation of piRNA biogenesis genes, which provide defense against transposons [18] to protect genome integrity. Germ cell progenitors harboring epimutations disrupting efficient male differentiation could permit transposon-based mutagenesis, leading to increased gamete diversity. Apoptosis, therefore, may balance the tradeoff between germ cell diversity versus epigenetic or genetic integrity. A key question is how extrinsic perturbations modulate the threshold of apoptosis and the rate of transposition.

Insights into how germline development resolves heterogeneity reflect a broader paradigm, whereby differentiation is an unstable process with stringent requirements to select for fitter cells with higher differentiation capability. It remains to be seen if similar principles guide selection in oogenesis, where heterogeneous differentiation can potentially nominate germ cell candidates to become oocytes versus doomed nurse cells. For example, in *Drosophila* oogenesis, asymmetric inheritance of cytoplasmic determinants across syncytial germ cells is critical for oocyte selection. New tools for single-cell analysis within germ cells could reveal their variance in female differentiation and its relation to cytoplasmic inheritance.

**Fig. 1:**
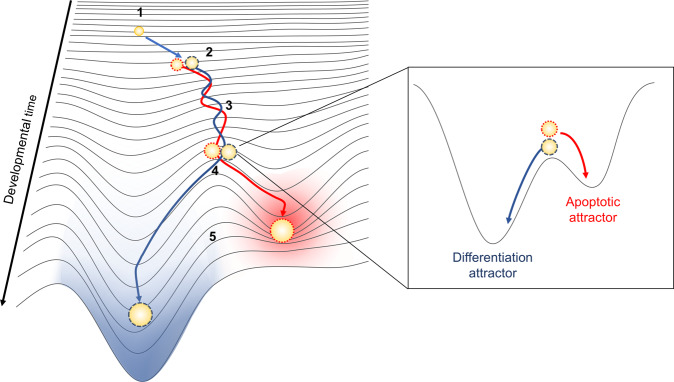
Instability during differentiation provides an opportunity for assessment of individual cellular phenotypes present in a heterogeneous differentiating population. In the above figure, a progenitor cell prior to differentiation is at a multipotent state (1). Acquisition of epimutations or other forms of heritable cellular diversity, as represented by dotted or dashed outlines (2), can alter the propensities of descendent cells toward attractor states. At the onset of differentiation (3), attractor states emerge, represented by depressions forming in the landscape. The adjacent presence of an apoptotic attractor state alongside a differentiation attractor state creates an unstable equilibrium (box). Differentiating cells proceed, balancing along this ridge until individual cellular phenotypes exert sufficient attraction toward either state (4), causing trajectories to diverge. Heterogeneous cells are consequently sorted into differentiated or apoptotic fates, with apoptotic cells terminating progress in an inescapable well (5).

Returning to Waddington’s landscape, rather than a multipartite ball rolling cohesively downwards, progenitor cells more accurately resemble a chaotically tumbling collection of craggy pebbles, each accumulating unique surface imperfections that induce instability in their progress. Cellular diversity necessitates a more complex model wherein progenitor cells adopt numerous trajectories, including elimination, should incurred epimutations disrupt differentiation (Fig. 1). Consequently, heterogeneity, differentiation, and apoptosis are three interrelated principles that together optimize formation of functional tissues from a dissimilar population of individual cells. How does cellular variation evolve over time? What strategies guide selection? Clonal tracing and single cell analysis techniques will be powerful tools for answering these questions and examining tissue biology through a new lens of cellular population dynamics.
